# Wettability control of polymeric microstructures replicated from laser-patterned stamps

**DOI:** 10.1038/s41598-020-79936-1

**Published:** 2020-12-30

**Authors:** Yangxi Fu, Marcos Soldera, Wei Wang, Stephan Milles, Kangfa Deng, Bogdan Voisiat, Kornelius Nielsch, Andrés Fabián Lasagni

**Affiliations:** 1 Institut Für Fertigungstechnik, Technische Universität Dresden, George-Bähr-Str. 3c, 01069 Dresden, Germany; 2 PROBIEN-CONICET, Dto.de Electrotecnia, Universidad Nacional del Comahue, Buenos Aires 1400, 8300 Neuquén, Argentina; 3 Institute for Metallic Materials, IFW Dresden, Helmholtzstr. 20, 01069 Dresden, Germany; 4 Institut Für Angewandte Physik, Technische Universität Dresden, Nöthnitzer Str. 61, 01187 Dresden, Germany; 5 Institut Für Werkstoffwissenschaft, Technische Universität Dresden, Helmholtzstr. 7, 01069 Dresden, Germany; 6 Fraunhofer-Institut Für Werkstoff- und Strahltechnik IWS, Winterbergstr. 28, 01277 Dresden, Germany

**Keywords:** Surface patterning, Polymers, Engineering

## Abstract

In this study, two-step approaches to fabricate periodic microstructures on polyethylene terephthalate (PET) and poly(methyl methacrylate) (PMMA) substrates are presented to control the wettability of polymeric surfaces. Micropillar arrays with periods between 1.6 and 4.6 µm are patterned by plate-to-plate hot embossing using chromium stamps structured by four-beam Direct Laser Interference Patterning (DLIP). By varying the laser parameters, the shape, spatial period, and structure height of the laser-induced topography on Cr stamps are controlled. After that, the wettability properties, namely the static, advancing/receding contact angles (CAs), and contact angle hysteresis were characterized on the patterned PET and PMMA surfaces. The results indicate that the micropillar arrays induced a hydrophobic state in both polymers with CAs up to 140° in the case of PET, without modifying the surface chemistry. However, the structured surfaces show high adhesion to water, as the droplets stick to the surfaces and do not roll down even upon turning the substrates upside down. To investigate the wetting state on the structured polymers, theoretical CAs predicted by Wenzel and Cassie-Baxter models for selected structured samples with different topographical characteristics are also calculated and compared with the experimental data.

## Introduction

Featuring low-density, flexibility, ease of processing and cost-effectiveness, polymers have been widely used in many different fields such as packaging, biomaterials, microelectronic devices and thin-film technology^1^. For instance, polyethylene terephthalate (PET) and poly(methyl methacrylate) (PMMA) have several attractive properties, including non-toxicity, high optical transmittance in the visible spectrum, and excellent mechanical and chemical properties. Furthermore, PET is widely used as a low-cost polymer in daily life with a broad range of applications, including food packages, containers, and even biomedical devices^2^. On the other hand, PMMA is an attractive material for replacing glass in many applications due to its lower weight and higher impact resistance. Additionally, PMMA is becoming a key material in microfluidics and biomedical fields for dental implants, bone cement, as well as cartilage replacement^2,3^. Inducing surface modifications on these polymers can provide new or better properties that could be appealing for new applications such as friction reduction, optoelectronic devices with enhanced efficiency, antibacterial surfaces, or holograms for anticounterfeiting^4–7^. Particularly, many innovative approaches designed to modify the wettability characteristics of polymer surfaces have been recently documented, paving the way for advanced liquid manipulation^8,9^. For instance, by coating silicone with a rough polymeric film, a superhydrophobic surface was created showing a very low ice-adhesion force^10^. Complex hierarchical topographies induced by colloidal lithography followed by plasma treatment allowed Ellinas and coworkers to produce PMMA surfaces with oil and water repellent characteristics, i.e. superamphiphobic, as well as surfaces that promote wetting of both of these test liquids, i.e. superamphiphillic^11^. In this direction, superhydrophobic (immersed in oil) and superoleophobic (in water) PMMA surfaces were fabricated by laser-etching, offering new possibilities for water–oil separation and enhanced oil recovery^12^. Furthermore, polymers with modified wettability characteristics have been used in the biomedical field. Namely, it was observed that neuronal and fibroblast cells have shown a preferential attachment on microstructured polydimethylsiloxane (PDMS) sheets with increased water contact angle, compared with the reference samples^13^.

It has been widely reported that lotus leaves and rose petals are two typical types of superhydrophobic surfaces designed by nature with opposite effects. For instance, the lotus leaves show low adhesion to water allowing droplets to easily roll off their surfaces, whereas rose petals present high adhesion preventing water droplets from moving at any tilted angles on the surfaces^14,15^. These particular wetting characteristics are not only attributed to the surface chemistry, but also to their nano- and micro-scale surface morphology. Therefore, surface texturing is often employed together with a chemical coating to modify the wettability of technical surfaces. Numerous techniques, such as hot/UV embossing^16,17^, photolithography^18–20^, and laser-based structuring^21,22^, have been utilized to engineer the surface topography on a large variety of materials.

For large scale production, hot embossing is one of the most used methods as it provides a low-cost, high-resolution, and high-throughput replication of structures at the micro- and nanoscale^23,24^ Due to the differences in the thermomechanical properties of the soft polymer and the hard mold, well-defined structures can be replicated from the mold to the polymers over large surfaces at one-step. Therefore, mold structuring plays a crucial role in achieving high-quality pattern replication. The most common methods to produce embossing molds include photolithography, micromachining, electroplating, etching, and laser structuring, among others^25–28^. Despite these methods are well-established, they still have some drawbacks, such as high cost, significant time consumption, multiple processing steps, limitation of feature resolution, and/or inaccurate dimension control^26,29,30^.

A one-step method providing high-resolution and low-cost processing for patterning hot-embossing molds is Direct Laser Interference Patterning (DLIP)^31^, by which a wide variety of periodic ordered patterns can be fabricated on the surfaces of various materials, including metals^32,33^, ceramics^34,35^, and polymers^36,37^. In this method, an interference pattern is transferred onto the sample surface by overlapping two or more coherent laser beams. At the intensity maxima positions, the material is directly ablated, thereby producing periodic patterns. Because of the interference phenomenon, a large number of periodic micro or sub-micro features can be generated after irradiating with a single laser pulse with an outstanding processing speed up to 1 m^2^ min^-1^^38,39^. In addition, this method offers the flexibility to adjust the spatial period (*Λ*) of the pattern by modifying the angle between the laser beams achieving typical lateral feature sizes from ~ 500 nm up to a few tens of micrometers^40^.

In this study, a simple and inexpensive approach is proposed to fabricate hydrophobic polymeric surfaces. With this strategy, periodic microstructures with a spatial period from 1.6 to 4.6 µm on a chromium (Cr) mold fabricated by DLIP were replicated onto PET and PMMA foils by using an electrohydraulic press. The static and dynamic wetting behaviors were studied to understand the effect of surface textures and surface roughness on the wetting performance of the two polymeric surfaces.

## Results and discussion

### Surface topography

**Figure 1:**
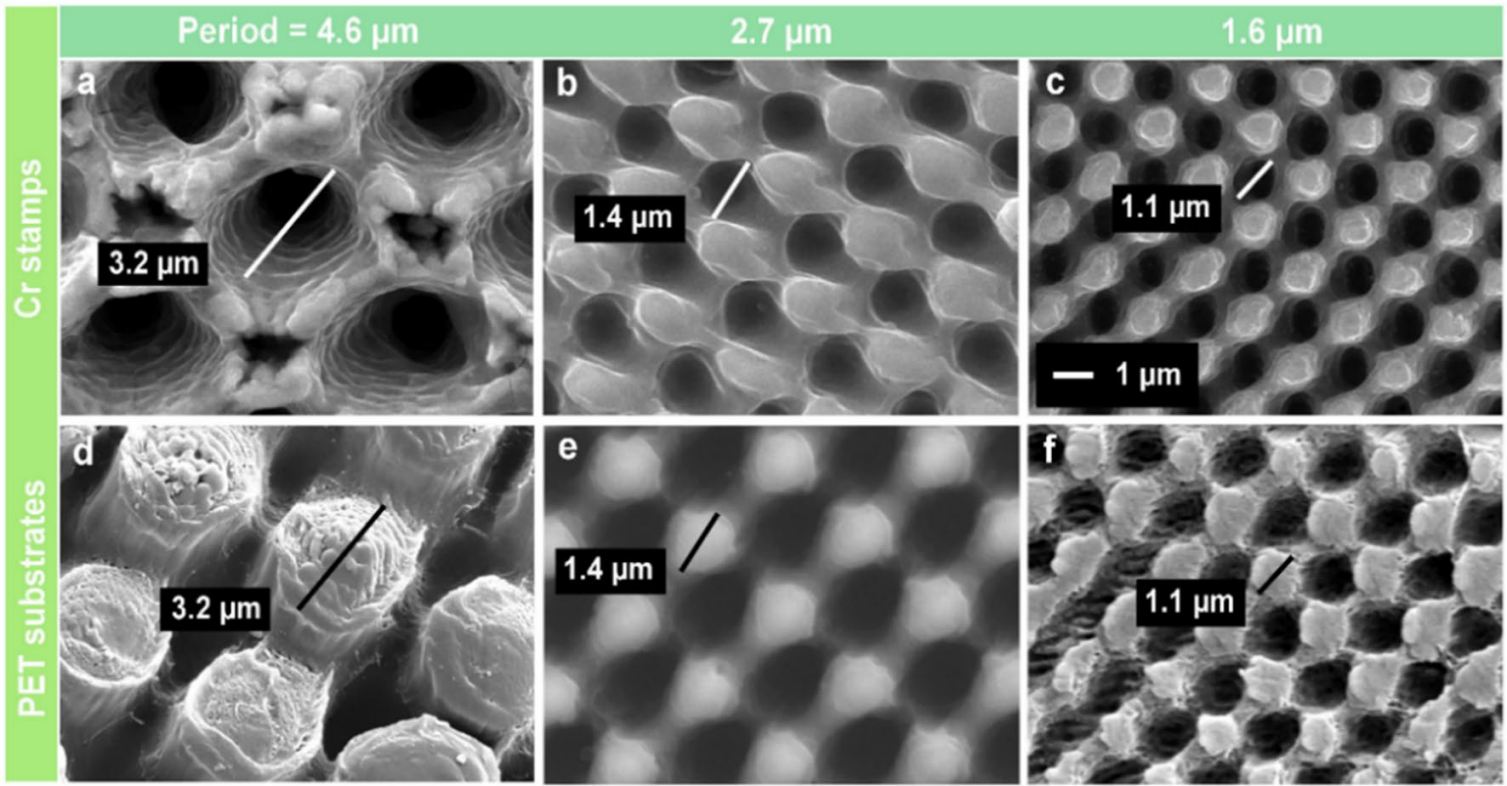
SEM micrographs of DLIP-produced hole-like periodic micropatterns on (**a**–**c**) chromium surfaces (stamp material) and pillar-like microarrays on (**d**–**f**) PET foils replicated from the stamps by hot-embossing. The spatial periods are: (**a**, **d**) *Λ* = 4.6 µm, (**b**, **e**) *Λ* = 2.7 µm and (**c**, **f**) *Λ* = 1.6 µm.

Periodic hole-like micropatterns were directly fabricated onto a Cr sheet by using four-beam DLIP in a one-step process. The morphology of the laser-treated Cr surfaces, shown in Fig. 1a–c, resembles regular ordered arrays of microholes formed after laser irradiation. By controlling the overlapping angle between the individual beams, the spatial period of the pattern arrays was set to (Fig. 1a) 4.6, (Fig. 1b) 2.7 and (Fig. 1c) 1.6 µm. Accordingly, the mean diameter of the craters also decreased from 3.2 to 1.1 µm. The rims of the craters raise above the surface and form conical protrusion arrays, which is attributed to the displacement of molten material around the holes^41,42^.

These produced periodic microhole arrays on Cr molds were replicated onto 200 µm thick PET and PMMA films by hot embossing. The SEM images of Fig. 1d–f show the surface morphology of PET replicas as an example. Uniformly distributed pillar-like structures with a period between 1.6 and 4.6 µm were successfully obtained. The size of replicated microstructures was nearly the same as that of the holes in the Cr mold, demonstrating an excellent fidelity to the Cr mold dimensions.

**Figure 2:**
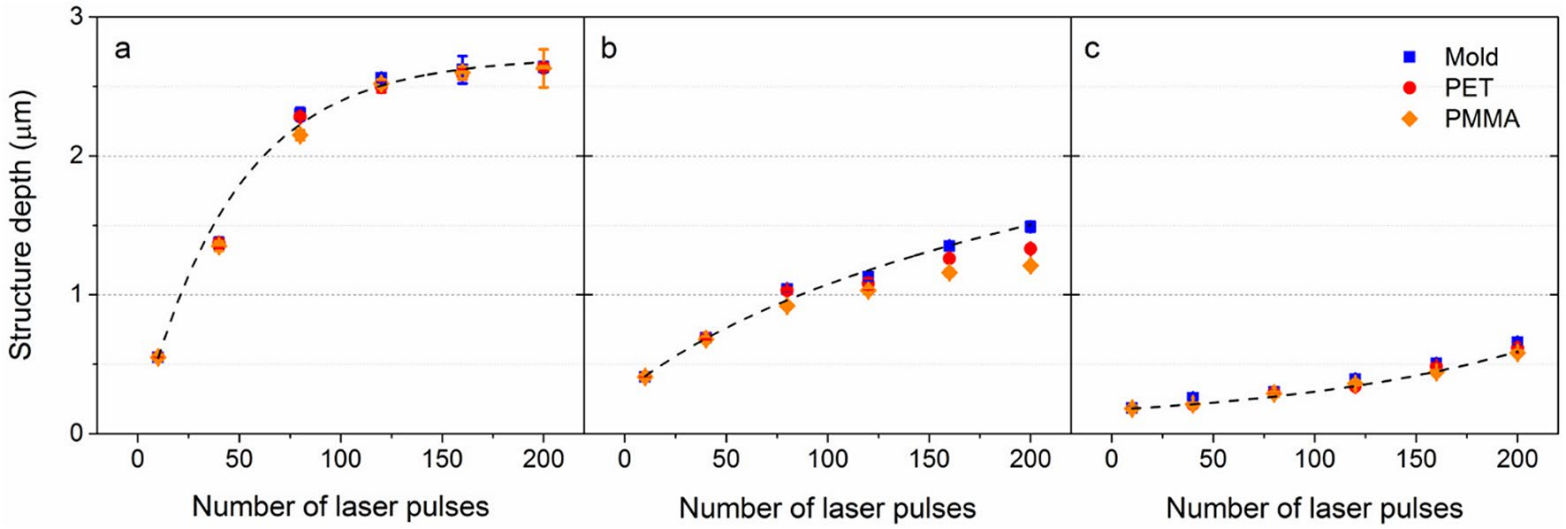
Evolution of structure height as a function of laser pulses for the spatial period of (**a**) *Λ* = 4.6 µm, (**b**) *Λ* = 2.7 µm and (**c**) *Λ* = 1.6 µm. The black dashed lines are guides to the eye.

The number of laser pulses *N* applied to the Cr sample was varied from 10 to 200 to gain a deeper insight into the influence of the laser processing conditions on the ablation efficiency and microstructures formation. The average depth of the laser-produced hole-like structures on Cr molds, and of the replicated pillar-like patterns on the polymers, were measured using confocal optical microscopy and are shown as a function of the number of laser pulses in Fig. 2. The results reveal a monotonic increase of the structure depths as the number of laser pulses increases, which can be well described by photo-thermal ablation behavior^43,44^. For the 4.6 µm period, the structure height tends to saturate after ~ 120 pulses (see Fig. 2a). This ablation depth saturation behavior has also been observed before^41^, which can be explained by recast material around the intensity maxima and multiple reflections in the keyhole evolution^45^. In addition, it can be observed that the structure depths for different periods differ significantly. For the larger interference period *Λ* = 4.6 µm, a maximum depth up to 2.6 µm was achieved, while for the 2.7 and 1.6 µm period, the maximum achieved depths were 1.5 and 0.62 µm, respectively.

After the hot embossing process, the heights of the pillar-like structures on PET and PMMA surfaces were also measured from their surface profiles. As shown in Fig. 2a, the mean structure height of the microfeatures with a period of 4.6 µm on PET and PMMA replicas are very similar to that on the corresponding molds (99.1% for PET and 98.2% for PMMA), indicating a complete filling of the soften polymers into the microcavities. On the other side, the maximum height of the pillars with a smaller period of 2.7 µm (see Fig. 2b) on PET and PMMA is 1.3 and 1.2 µm, respectively, compared to the structure depth of 1.5 µm on the Cr surface irradiated with 200 laser pulses (86.7% and 80.0% for PET and PMMA, respectively). This difference in structure height of plastic substrates and Cr mold is caused by incomplete filling during the hot embossing process, probably due to smaller cavities limiting the softer polymer to flow into them. This deviation in the height of the embossed structures from the mold is only observed on the 2.7 µm period samples irradiated with a higher number of pulses (> 150 pulses). In addition to the structure height, the aspect ratio (*AR*), defined as the quotient between the height and period of the micropillars (*AR* = *depth*/Λ), is used to describe the surface topographies. By increasing the number of laser pulses, the $AR$ of the micropillars with a period of 4.6 µm increases from 12 to 57% on both PET and PMMA. Differently, *AR* up to 49% was observed for the 2.6 µm-period, while for the 1.6 µm patterns, the *AR* is smaller than 40%.

### Static wetting behavior

**Figure 3:**
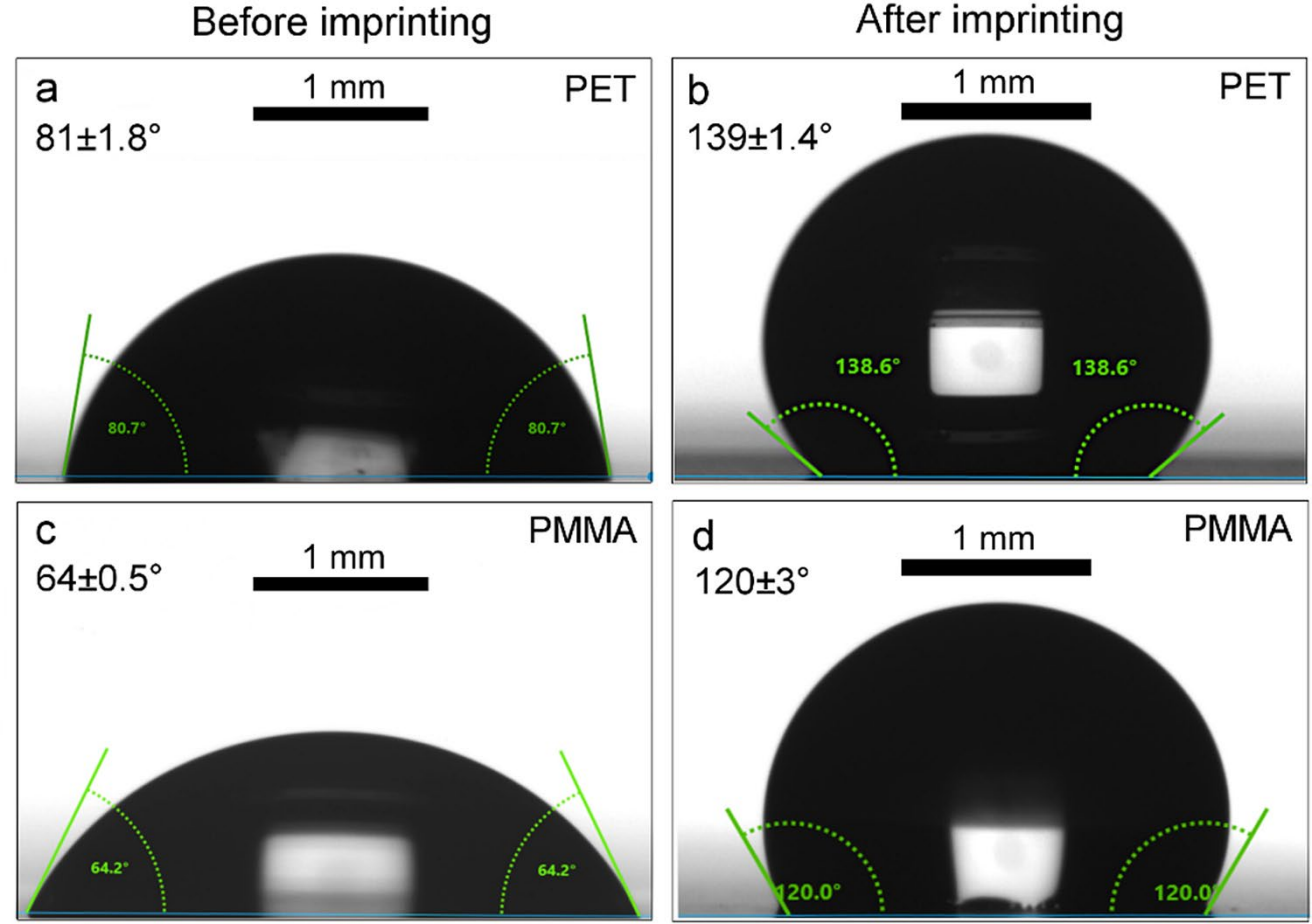
Water droplets with a volume of 7 µl on (**a**) flat and (**b**) an imprinted PET film (pillar-like structures with a period of 4.6 µm and a depth of 2.6 µm) with water CA of 81 ± 1.8° and 139 ± 1.4°, respectively; water droplets on (**c**) flat PMMA and (**d**) imprinted PMMA film (pillar-like structures with a period of 4.6 µm and a depth of 2.6 µm) with water CA of 64 ± 0.5° and 120 ± 3°, respectively.

To evaluate the wettability of the imprinted polymeric surfaces, the static water contact angle of 7 µl-water droplets on of the PET and PMMA samples was measured before and after the hot embossing step. As shown in Fig. 3, the static contact angle of untreated flat PET (Fig. 3a) and PMMA (Fig. 3c) is 81 ± 1.8° and 64 ± 0.5°, respectively, demonstrating a hydrophilic behavior of pristine PET and PMMA without any surface texturing or chemical modification. After the periodic microstructures consisting of pillars with a depth of 2.6 µm and distributed with a period of 4.6 µm were replicated by hot embossing at 85 °C for 5 min, the wettability of PET is turned to a hydrophobic state with a maximum CA up to 139 ± 1.4° (Fig. 3b). Likewise, pillar-like features with exactly the same periodicity and depth imprinted onto PMMA films induced an increase in the CA to 120 ± 3° (Fig. 3d). These results demonstrate that the imprinted periodic microfeatures are capable to significantly increase the static CA on PET (or PMMA) for over 71% (or 88%) compared to its flat reference surfaces without any chemical modification.

**Figure 4:**
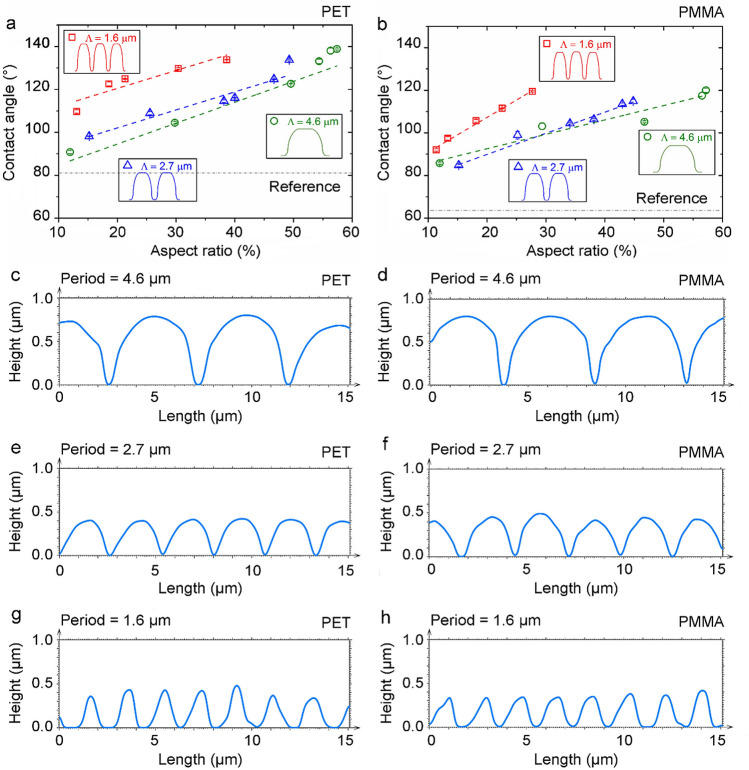
Static contact angle of water as a function of the aspect ratio of the imprinted pillar-like structures on (**a**) PET and (**b**) PMMA. The reference (grey) lines correspond to the water contact angle on the smooth original polymer surfaces, whereas the colored dashed lines represent linear fits to the data. Topographical profiles of (**c**, **e**, **g**) patterned PET and (**d**, **f**, **h**) PMMA with a spatial period of (**c**, **d**) 4.6 μm, (**e**, **f**) 2.7 μm, and (**g**, **h**) 1.6 μm, respectively.

To study the effect of surface topography on the wetting characteristics of plastic substrates, the measured CAs on patterned PET and PMMA surfaces were plotted as a function of the aspect ratio for different spatial periods ranging from 1.6 to 4.6 µm (Fig. 4a,b). The results show that the contact angle on both polymers presents an upward trend with the aspect ratio, independently of the structure period. In the case of the patterns with a period of 4.6 µm, the CAs on PET range from 91 ± 2° to 139 ± 1.4° as the aspect ratio increases from 12 to 57%. A similar trend was also observed for the patterns with a spatial period of 1.6 and 2.7 µm, although the maximum aspect ratio for the 1.6 and 2.7 µm-period is only 39% and 49%, respectively. However, it is worth noting that the periodic patterns with the smallest spatial periods of 1.6 µm show a higher contact angle than the surfaces with a spatial period of 2.7 and 4.6 µm, for comparable values of aspect ratios. A similar trend was also observed on the imprinted PMMA surfaces. In this case, for the 2.7 µm and 4.6 µm periodic surface patterns with a height of 0.41 and 0.55 µm, respectively, the CAs do not exceed 90° indicating a hydrophilic surface, although the CAs were increased by 33% compared to its flat reference. However, in case of the 1.6 µm period, a hydrophobic surface with a mean CA of 92 ± 1.8° can be achieved even with features with a low structure height of 0.18 µm. The periodic patterns with the smallest spatial period (*Λ* = 1.6 µm) also present higher contact angles compared to the larger ones, which is consistent with the result observed on imprinted PET (Fig. 4a).

Similar results were reported on polyurethanes (PU) structured directly by DLIP method with line-like patterns featuring spatial periods between 2 and 5 µm^46^. The results showed that the structures with 2 − 3 µm periods present higher contact angles compared to the patterns with 4 – 5 µm. This effect was explained by the differences in the surface topography of the structured samples. In the present experiments, the surface profiles in Fig. 4c–h also show that the periodic microfeatures on PET and PMMA have different topography shape for different periods. For instance, the profiles with *Λ* = 2.7 µm and *Λ* = 4.6 µm (Fig. 4c–f) exhibit a shape similar to half-spheres with narrow trenches separating them, which yields a relatively large area where the water droplets can sit. In contrast, the samples with the smallest period (Fig. 4g,h) have a triangular-like profile with sharp peaks and thus with a smaller droplet seating area. This evident different profile shape might cause the difference in the wetting behavior of micropatterns with similar aspect ratio but different spatial periods.

As described above, surface topography on the polymeric surfaces significantly influences the wettability, because the changes in the texture pattern and roughness cause variations in the actual liquid–solid contact area. In general, two models are used to describe the interactions of liquids and solids with textured surfaces: (1) the Wenzel model, when the liquid fills the valleys of a rough solid surface; and (2) the Cassie-Baxter model, when air is trapped inside the valleys between the drop and the surface^47,48^. Wenzel model describes the variation of water contact angle according to Eq. (1),

1 $$\cos \theta _{w} = W\cos \theta _{0} ,$$

where *θ*_w_ is the CA of the rough surface, *W* is the roughness factor defined as the ratio between the real surface area in contact with the liquid and its projected area, and *θ*_0_ is the CA measured on a flat surface. The Wenzel model then predicts that an originally hydrophilic surface becomes more hydrophilic as the surface roughness increases, whereas a hydrophobic character increases when the starting surface is hydrophobic. In the case of the Cassie-Baxter model, the contact angle *θ*_c_ is described by:

2 $$\cos \theta _{c} = C\cos \theta _{0} - (1 - C),$$

where*θ*_0_ is the reference contact angle of a flat surface, *C* is a roughness parameter based on the ratio between the solid–liquid line and its projected area. According to Eq. (2), the increase of surface roughness leads to an increase in the hydrophobic character regardless of whether the original flat surface is hydrophilic or hydrophobic.

**Figure 5:**
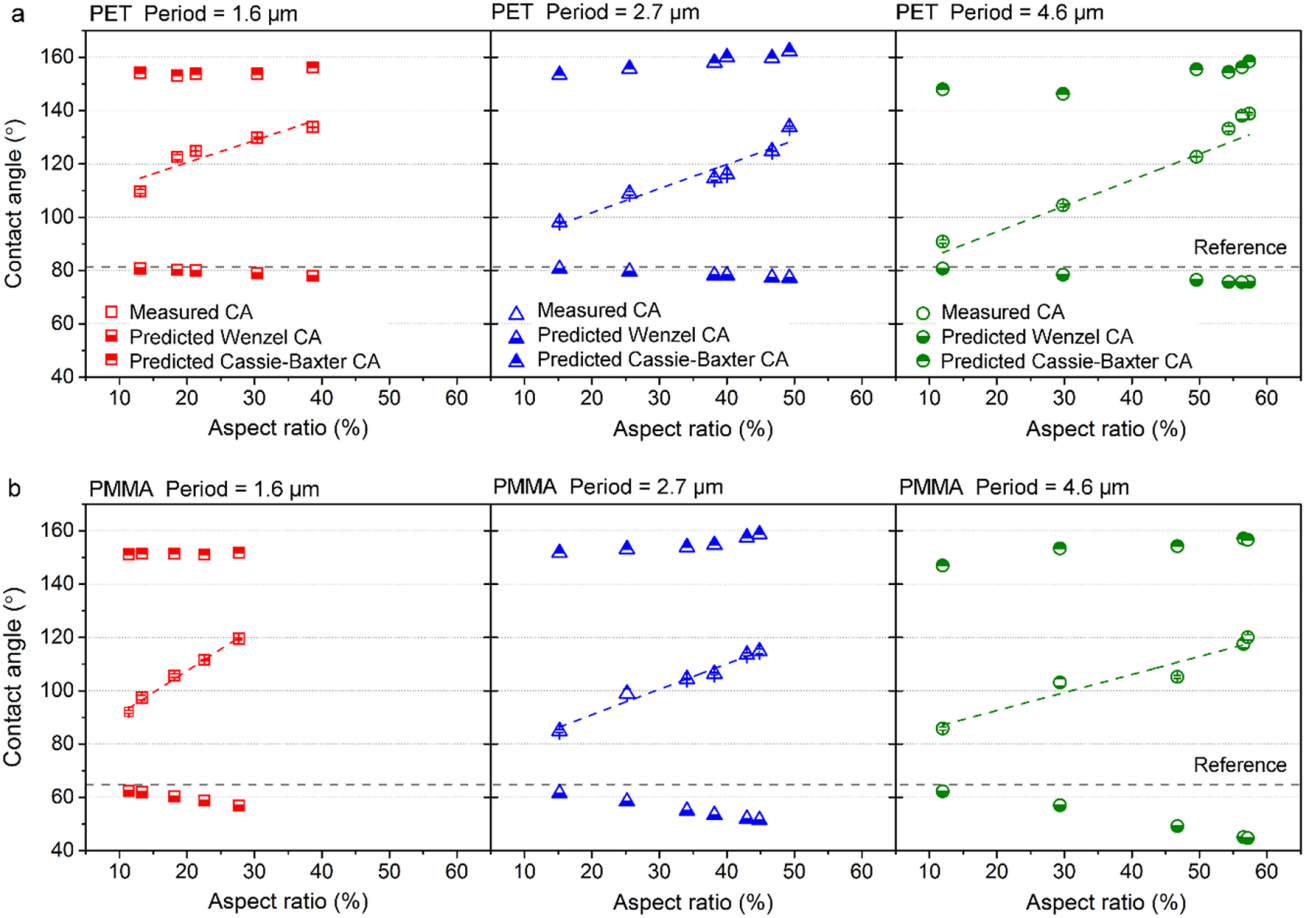
Comparison of measured and predicted water contact angles as a function of aspect ratio of the imprinted pillar-like structures on (**a**) PET and (**b**) PMMA. The reference lines correspond to the water contact angle on the smooth original polymer surfaces.

The models of Wenzel and Cassie-Baxter can be used to calculate the contact angles *θ*_w_ and *θ*_c_ according to Eqs. (1) and (2) for better understanding the influence of the topographical characteristics in the wetting state of water droplets in contact with polymer surfaces. The detailed calculations can be found in the supplementary information. For comparison, Fig. 5a,b present the measured and calculated CAs as a function of surface texture aspect ratio for PET and PMMA substrates, respectively. In the case of PET, the predicted Wenzel angles *θ*_w_ varies from 76° to 81° depending on the surface aspect ratio and the spatial period, while the Cassie-Baxter contact angles *θ*_c_ show a hydrophobic wetting state ranging from 146° to 162°. Regarding PMMA, the *θ*_w_ is between 45° and 62°, while the *θ*_c_ ranges from 147° to 159°.

Besides, a significant discrepancy between the experimentally measured *θ* and the calculated *θ*_w_ and *θ*_c_ angles can also be observed. Furthermore, for both materials the measured contact angles *θ* lie between the CAs calculated with the models, and they increase with the surface aspect ratio. The results also show that the wetting state depends on further topographical characteristics of the imprinted microstructures, such as spatial period and depth. For example, for lower aspect ratios ~ 0–10% and in particular for larger spatial periods (e.g. 4.6 µm), the experimental CAs for both materials are closer to the predicted Wenzel CAs. However, clearly the measured contact angles *θ* do not show a decreasing trend as the Wenzel (*θ*_w_) condition. Differently, for higher aspect ratios, the measured CAs are better described by the Cassie-Baxter condition. For example, the periodic structure produced on PET with *Λ* = 4.6 µm and *AR* = 57% (rightmost image of Fig. 5a), has a measured contact angle of 139 ± 1.4°, which is closer to the predicted Cassie-Baxter angle of 158° (compared to the corresponding Wenzel angle of 76°). Thus, deeper structures seem to be more efficient to prevent the water drops from penetrating into the structure valleys. Similar is the case for PMMA, where the highest CA of 120°, obtained for a pattern with the same aspect ratio, was only 24% lower than the calculated Cassie-Baxter angle (*θ*_c_ = 157°).

According to previous results^49,50^, pure Cassie-Baxter or Wenzel wetting situations rarely occur, as the wettability of real surfaces is much more complicated. Since our experimental CAs lie between the Wenzel and Cassie-Baxter calculated angles, it can be assumed that the rough surfaces produced on both PET and PMMA adopt neither Wenzel nor Cassie-Baxter states, but an intermediate state. Many researchers have observed this behavior on microstructured surfaces as well, which is called composite or mixed wetting state^49–53^. In this state, the water droplet partly sits on air pockets as well as it partly penetrates the valleys of the rough solid surface. For example, He et al. measured a composite wetting state on a rough PDMS surface patterned with perfect square pillar arrays^52^. In this case, the measured contact angle of 152.5°, lies between the theoretical calculated Cassie-Baxter (165.7°) and the Wenzel angles (121°).

The reason for the composite wetting is still under discussion. According to Whyman et al., wetting of inherently hydrophilic surfaces is always energetically favorable, while Cassie-Baxter wetting is possible only when air is trapped into the rough surfaces^54^. Therefore, patterned superhydrophobic surfaces sometimes lack stability on Cassie-Baxter wetting state due to the low energy barrier separating the Cassie-Baxter and the Wenzel states. Lafuma et al. reported that the mixed state usually happens when the surface is decorated with a single pattern, which is not very rough, and its hydrophobicity is moderate (e.g. θ = 110°)^55^. Therefore, the Cassie-Baxter state of air trapping is metastable and irreversible transitions towards other states can be easily induced. However, recent studies reported that this composite wetting also exists on some complicated hierarchical surfaces, such as in rose petals^49,56^. Wetting transitions can also occur spontaneously or induced by external stimuli such as gravity, pressure, vibration, or bouncing of the droplet^49,54^. As the laser-induced surface morphology is typically complex, which usually lacks homogeneity due to the Gaussian profile of laser beams or due to additional surfaces features (e.g. nanoscaled laser-induced periodic surface structures (LIPSS))^31^, it is difficult to obtain an accurate characterization of the microscopic wetting state. Therefore, for a better understanding of the wetting phenomenon at the microscale level, further investigation needs to be done in the future.

### Dynamic wetting behavior

**Figure 6:**
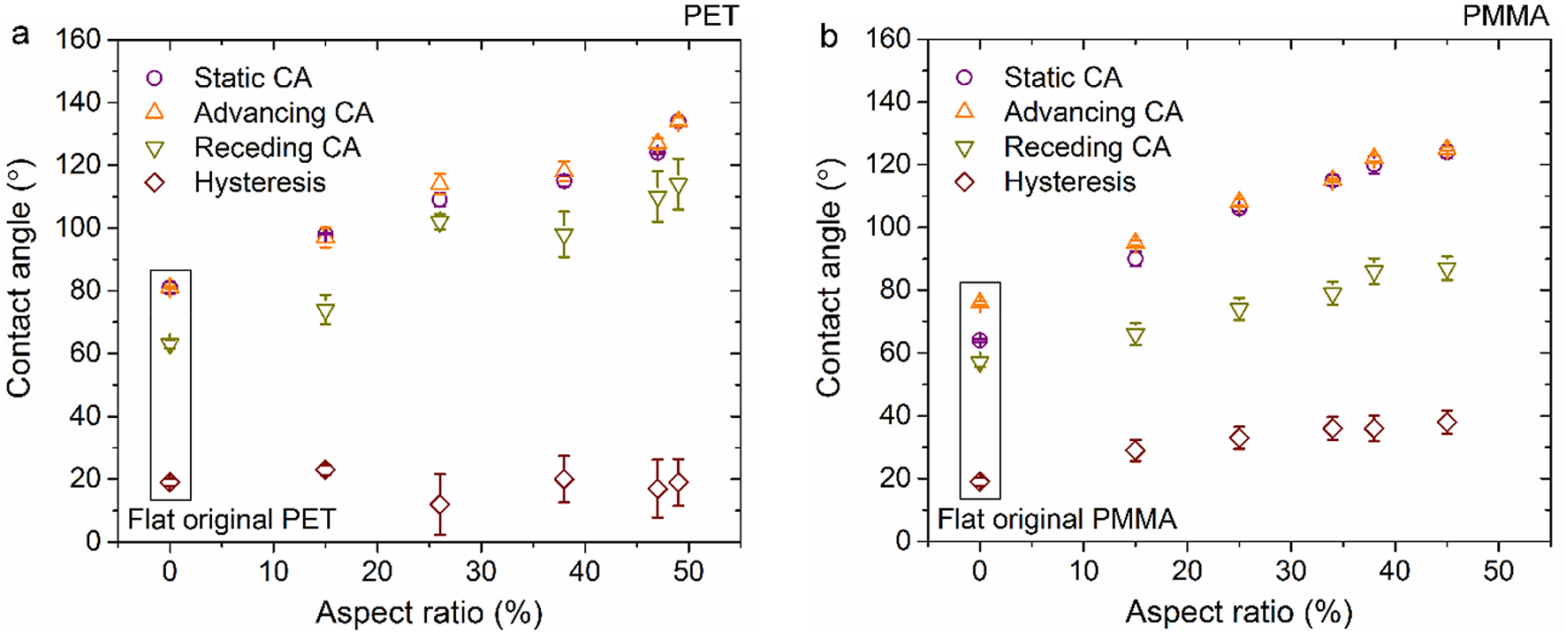
Static contact angles, advancing/receding contact angles, and hysteresis for water on flat and structured (**a**) PET and (**b**) PMMA with various texture aspect ratio and fixed period of 2.6 µm.

To further investigate the wetting characteristics of the imprinted polymers, the dynamic contact angles of water on textured PET and PMMA with a periodicity of 2.6 μm were determined as shown in Fig. 6. Only one period is shown here since the results show negligible dependence on the period compared to the surface aspect ratio. The contact angle hysteresis *Δθ* is defined as the difference between the advancing *θ*_a_ and receding *θ*_r_ contact angles^57^:

3 $$\Delta \theta = \theta _{a} - \theta _{r} ,$$

It can be seen in Fig. 6, that the advancing contact angles of water droplets on flat PET and PMMA surfaces are 81 ± 0.4° and 76 ± 0.6°, respectively, while the receding angles are 63 ± 1.3° and 57 ± 1.5°. The average hysteresis *Δθ* on PET and PMMA was calculated using the Eq. 3, obtaining the same value of 19° for both materials. H. Y. Erbil et al. reported receding contact angles *θ*_r_ = 64°—66° for PET and *θ*_r_ = 54°—64° for PMMA with an average hysteresis of 19.5 ± 1.5° (PET) and 23.5 ± 1.5° (PMMA)^58^, respectively, which is consistent with our measurements.

Regarding the textured PET and PMMA surfaces, the results show that both the advancing and receding angles increase with the texture aspect ratio. The hysteresis *Δθ* for textured PET varies in the range between 12 ± 9.6° and 23 ± 1.6° without showing a clear trend with the aspect ratio of the microstructure. In case of PMMA, a slight increase of the hysteresis was observed as a function of the aspect ratio, reaching values up to 38 ± 3.7°. According to the previous report by K.-Y. Yeh et al., when a droplet on a patterned surface falls into the Wenzel state, the advancing contact angles increase with an increase in the surface roughness, whereas the receding angle decrease^59^. On the other hand, if a droplet on a surface belongs to Cassie-Baxter state, the advancing and receding contact angles are independent of the surface roughness. In addition, they also reported that hysteresis *Δθ* remains constant independently of the surface roughness values. As observed in Fig. 6, any of these two wetting behaviors are consistent with our findings. Discontinuity of the receding contact angle and the contact angle hysteresis can be observed in Fig. 6, which further confirmed that the structured PET and PMMA surfaces adopt a composite wetting state.

The contact angle hysteresis can be used for estimating a measure of the “stickiness” of a surface^60^. D. Quéré et al.^57^ calculated the droplet sticking condition according to the numerical calculation of the sliding of a liquid drop on a solid surface^61^:

4 $$\pi \ell \sigma (\cos \theta _{r} - \cos \theta _{a} ) = \rho g\varsigma \sin \gamma ,$$

where *l* is the radius of a droplet which is in contact with a substrate, *σ* is the liquid/vapor surface tension, *ρ* is the density of the liquid, *g* is the gravitational acceleration, *ζ* is the drop volume (*ζ* = *4π/*3*R*_*0*_^3^), *R*_0_ is the radius of curvature of the droplet, and *γ* is the critical angle of inclination. By introducing an average angle *θ* (*θ*_*r*_ = *θ—Δθ*/2 and *θ*_*a*_ = *θ* + *Δθ*/2), the drop will remain stuck, when:

5 $$\Delta \theta \ge \Delta \theta _{c} = \frac{{4^{{2/3}} }}{3}(R_{0} \kappa )^{2} \frac{{(2 + \cos \theta )^{{1/3}} (1 - \cos \theta )^{{2/3}} }}{{\sin ^{2} \theta }}\sin \gamma ,$$

where *κ* ^-1^ is the capillary length, which is in the range of millimeters for most systems^57^. Small droplets (*R*_0_ < *κ* ^-1^) will stick when the substrate is inclined by a moderate angle, whereas a droplet that is significantly larger than the capillary length will move down because of the gravity^57^. For a 7 µl-droplet with a radius *R*_0_ of 1.19 mm, the drop is found to roll only when *Δθ* < 4 – 5° or *θ* > 160°; otherwise, it will stick to the surface^57^. However, when the tilting angle of the substrates is larger than the angle of sliding (or angle of repose), the droplet will move down.

**Figure 7:**
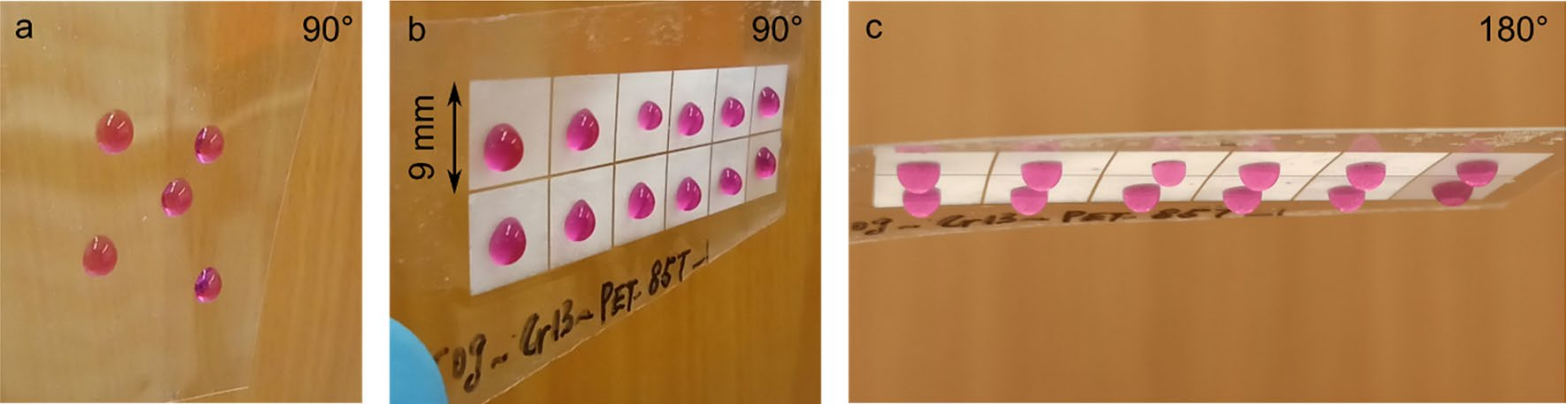
Photographs of water droplets (dyed by 0.1% KMnO_4_ solution) on (**a**) a flat and imprinted PET film with a tilting angle of (**b**) ~ 90° and (**c**) ~ 180°. See text for processing parameters of each structured area.

An impression of the stickiness of water on the flat hydrophilic and structured hydrophobic PET surfaces can be grasped by looking at the photographs in Fig. 7. For better visualization, water was dyed by 0.1% KMnO_4_ solution to create a colorful contrast. As it can be seen in Fig. 7a, the water droplets slide down on smooth untreated PET surface when the foil is tilted to a vertical position. On the contrary, the drops preserve their spherical cap geometries on the imprinted PET foil in each of the 9 × 9 mm^2^ textured areas with pillar-like microstructures (Fig. 7b). The laser-patterned stamp was processed with different parameters corresponding to each of the structured areas. Namely, the structured areas on the top row of the imprinted PET sample have a constant period of 1.6 µm, while the bottom areas have a texture spatial period of 4.6 µm. From right to left, the number of applied pulses was varied in the following sequence: 10, 40, 80, 120, 160, and 200. Furthermore, the droplets do not slide down when the sample is tilted even by 90°. Figure 7c shows droplets remaining immobile and stuck to the microstructured areas on PET even when the sample is turned upside down (rotated by 180°), independently of the surface roughness. These results confirm a high adhesion of water to the structured surfaces, and a negligible influence of the surface roughness on the stickiness. Similar results were also observed on PMMA surfaces.

Surfaces with a high contact angle and very strong adhesion to wetting fluid are termed parahydrophobic surfaces^62^. Rose petal is a prime example exhibiting this parahydrophobic behavior, known as “rose petal effect”. The reason for the unique adhesive superhydrophobicity in this natural example is its special hierarchical microstructure, which is composed of periodic micropapillae arrays of 16 μm diameter and nanoscale cuticular folds of 730 nm width, decorating the top of the micropapillae^15^. The wetting state of water on rose petals was recently determined using confocal microscopy, showing that water does not fully wet all the surface features, but only partially wets the gaps between micropapillae in a composite wetting^56^. Likewise, the composite wetting is assumed to be the reason for the adhesive hydrophobic surfaces in this study.

## Conclusions

In this study, we have reported a rapid approach for producing periodic pillar-like microarrays on PET and PMMA foils by utilizing Direct Laser Interference Patterning combined with the hot embossing method. Under optimized processing conditions, micropillar arrays with spatial periods from 1.6 to 4.6 µm and structure heights between 180 nm and 2.6 µm were successfully fabricated. The measured surface aspect ratio of micropillar arrays ranged between 12 and 57%. Static water contact angle measurements of the PET and PMMA replicas confirmed that the micropillar arrays can increase CAs leading to hydrophobic surfaces. The theoretical predictions of CAs were compared to systematic matching experiments. The results showed that the measured CAs lied between the calculated Wenzel and Cassie-Baxter CAs, indicating that a composite wetting behavior occurred on the rough surfaces. Both advancing and receding angles were found to increase with the surface roughness on both materials. The contact angle hysteresis slightly varied between 12° and 23° for PET and between 29° and 38° for PMMA. Furthermore, the water droplets presented a high adhesion to the polymer surfaces, which indicates that further modifications of the polymer surfaces are necessary to reach the super-hydrophobic conditions, which will be investigated in the future.

## Experimental section

### Materials

PET polymer foils were purchased from Pütz Folien GmbH + Co. KG and PMMA foils (PLEXIGLAS@) from Evonik Performance Materials GmbH. According to the manufacturers, PET and PMMA have glass transition temperatures (*T*_g_) of 85 °C and 109 °C, respectively. The thickness of both PET and PMMA were 200 µm, which were much thicker than the deepest structure formed. The used mold consisted of a chromium (Cr) sheet with a size of 87.4 × 66.7 mm^2^ and a roughness (*R*_a_) of 50 nm, provided by Sächsische Walzengravur GmbH.

### Picosecond-direct laser interference patterning

A self-developed DLIP workstation (VIS/IR-DLIP μFAB, Fraunhofer IWS, TU Dresden) consisting of a solid-state picosecond-pulsed laser system (NeoLASE GmbH) with a wavelength of 532 nm and a pulse duration of 70 ps was utilized for texturing the Cr mold by overlapping four Gaussian beams (TEM00 mode) on the mold surface. The angle between the laser beams was varied to obtain hole-like structures with spatial periods of 1.6, 2.7 and 4.6 µm. The evolution of the topography on the Cr surfaces was studied as a function of the applied number of pulses N, namely 10, 40, 80, 120, 160, and 200 pulses at a constant fluence of 3.5 J cm^-2^ and a fixed repetition rate of 10 kHz. A detailed description of the used setup and the fabrication steps can be found elsewhere.^31^.

### Plate-to-plate hot embossing

The as-prepared chromium sheets were utilized to replicate the laser-produced micropatterns on PET and PMMA films by using an electrohydraulic press (Paul-Otto Weber GmbH). The hot embossing process involves four steps^31^. First, the polymer foil was placed between two heated plates at a temperature around the glass transition temperature of the polymer, which is 85 °C for PET and 115 °C for PMMA. Next, the polymer foil was compressed with a force of 200 kN, yielding a pressure of 34.3 MPa, for 5 min, aiming to force the polymer to fill the cavities of the Cr mold. Afterward, the mold and plastic substrate were cooled down to 40 °C while maintaining the pressure constant for 5 min. Finally, the embossing components were demolded at room temperature by releasing the plastic substrate from the mold. No additional anti-sticking layer was applied on the Cr mold prior to embossing.

### Surface characterization

High-resolution scanning electronic microscopy (Phillips XL30 ESEM-FEG, JEOL JSM-6510 and Zeiss Sigma 300) was utilized to visualize the surface morphology of the mold and the imprinted polymers. The polymer samples were sputtered with a 20 nm gold layer before the SEM measurement for providing electrical conductivity at the material surface. Surface topography of the patterned microstructures was determined using a confocal optical profiler (Sensofar S neox) with an 150 × objective, which provides lateral and vertical resolutions of 140 nm and 1 nm, respectively. The measured 3D profiles were processed by SensoMap software to calculate the structure depth and periodicity of the patterns^31^.

### Contact angle measurement

Static and dynamic water contact angle measurements were performed on a drop shape analyzer (DSA100S, KRÜSS GmbH) in an ambient environment of 25 °C temperature and 16% of relative humidity. The needle diameter was 0.519 mm, and the water droplet volume was set to ~ 7 µL. Regarding the static contact angle measurement, the droplet shape was fitted with the Young–Laplace drop profile fitting method. The contact angle hysteresis, as well as the advancing/receding contact angles, were measured using the needle method by injecting/withdrawing liquid to/from the existing droplet at a constant volume flow rate of 1.0 µL s^-1^. Each measurement was repeated 4 times for a better statistical significance. The error bars resulted from the standard deviation based on the measurement series.

## Supplementary information

Supplementary Information 1.
