# Supplementary material for: Wettability control of polymeric microstructures replicated from laser-patterned stamps
Source: Sci Rep. 2020 Dec 30;10:22428. doi: 10.1038/s41598-020-79936-1 (PMC7773741; doi:10.1038/s41598-020-79936-1)
Supplement: Supplementary file 1 — Supplementary Information 1. [file 41598_2020_79936_MOESM1_ESM.pdf]

Supplementary Information

Wettability control of polymeric microstructures replicated from laser-patterned stamps

Yangxi Fu, Marcos Soldera, Wei Wang, Stephan Milles, Kangfa Deng, Bogdan Voisiat, Kornelius Nielsch, Andrés Fabián Lasagni*

The imprinted pillar-like periodic micropatterns on the polymeric substrates can be considered as uniformly distributed truncated cone arrays as shown in Figure S1. For this geometry, the following relationship can be obtained between the spatial period Λ and the geometrical parameter d, defined as the distance between the consecutive cones:

$$d^2 + d^2 = (2\Lambda)^2 \rightarrow d = \sqrt{2}\Lambda \quad \text{(S1)}$$

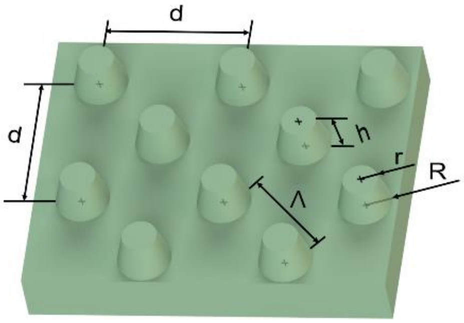

Figure S1. 3D representation of the imprinted micropatterns on PET and PMMA.

Then, the area of the exterior wall of the truncated cones F can be defined as:

$$F = \pi R\sqrt{R^2 + \left(h + \frac{hr}{R-r}\right)^2} - \pi r\sqrt{r^2 + \frac{(hr)^2}{(R-r)^2}} = \pi(r+R)\sqrt{h^2 + (R-r)^2} \quad \text{(S2)}$$

where R is the radius of the cone bases, r is the radius of the cone truncated bases, and h is the structure height.

In the case of the Wenzel model, the liquid is in contact with the whole surface. Therefore, the Wenzel constant can be calculated as:

$$W = \frac{d^2 - 2\pi R^2 + 2\pi r^2 + 2F}{d^2} = 1 + \frac{2\pi(r+R)\left(\sqrt{h^2+(R-r)^2} - (R-r)\right)}{2\Lambda^2} \quad \text{(S3)}$$

Differently, the liquid only is in contact with the truncated bases in the case of the Cassie-Baxter model. Therefore, the Cassie-Baxter constant W can be calculated as:

$$C = \frac{2\pi r^2}{d^2} \quad \text{(S4)}$$

Using the constants W and C from Equations S3 and S4, the theoretical Wenzel θ_w and Cassie-Baxter contact angle θ_c can be determined for each structure. A summary of the topographical parameters h, r, R and Λ derived from the measured surface structures as well as the measured and calculated contact angles for both imprinted PET and PMMA materials are listed in Table S1.

Table S1. Topographic parameters and measured and calculated CA for PET and PMMA materials.

Pattern Nr.	Polymer	Topographical parameters				CA predicted		CA measured
		h [µm]	r [µm]	R [µm]	Λ [µm]	θ_w [°]	θ_c [°]	θ_m [°]
1	PET	0.55	0.94	1.90	4.60	80.75	147.97	90.79
2	PET	1.37	0.99	1.75	4.60	78.39	146.28	104.46
3	PET	2.28	0.73	1.93	4.60	76.49	155.46	122.63
4	PET	2.50	0.76	1.96	4.60	75.68	154.45	133.14
5	PET	2.59	0.71	1.95	4.60	75.60	156.21	138.00
6	PET	2.64	0.64	1.82	4.60	75.75	158.43	138.84
7	PET	0.41	0.46	1.14	2.70	80.60	153.38	98.14
8	PET	0.69	0.42	1.10	2.70	79.62	155.70	108.90
9	PET	1.03	0.38	1.02	2.70	78.23	157.97	114.55
10	PET	1.08	0.35	1.11	2.70	78.18	159.92	116.07
11	PET	1.26	0.35	1.04	2.70	77.32	159.67	124.79
12	PET	1.33	0.31	1.11	2.70	77.23	162.23	133.72
13	PET	0.21	0.27	0.65	1.60	80.76	154.06	109.68
14	PET	0.30	0.28	0.63	1.60	80.23	153.07	122.54
15	PET	0.34	0.27	0.61	1.60	79.95	153.77	124.89
16	PET	0.49	0.27	0.65	1.60	78.94	153.68	129.76
17	PET	0.62	0.25	0.56	1.60	77.98	156.08	133.82
18	PMMA	0.55	0.87	2.00	4.60	62.28	146.93	85.81
19	PMMA	1.35	0.70	1.94	4.60	57.01	153.41	103.12
20	PMMA	2.15	0.69	2.03	4.60	49.20	154.16	105.19
21	PMMA	2.60	0.61	1.88	4.60	45.08	157.06	117.49
22	PMMA	2.63	0.62	1.76	4.60	44.69	156.60	120.05
23	PMMA	0.41	0.44	1.08	2.70	61.56	151.71	84.86
24	PMMA	0.68	0.42	1.08	2.70	58.44	153.12	98.95
25	PMMA	0.92	0.41	1.07	2.70	55.00	153.84	104.43
26	PMMA	1.03	0.39	1.06	2.70	53.38	154.66	106.37
27	PMMA	1.16	0.35	1.09	2.70	51.99	157.47	113.56
28	PMMA	1.21	0.33	1.06	2.70	51.49	158.68	114.83
29	PMMA	0.18	0.27	0.60	1.60	62.41	151.14	92.03
30	PMMA	0.21	0.26	0.60	1.60	61.94	151.42	97.42
31	PMMA	0.29	0.26	0.55	1.60	60.35	151.3	105.67
32	PMMA	0.36	0.27	0.57	1.60	58.81	151.03	111.55
33	PMMA	0.44	0.26	0.56	1.60	56.93	151.69	119.46
